# Reduction in parvalbumin expression not loss of the parvalbumin-expressing GABA interneuron subpopulation in genetic parvalbumin and shank mouse models of autism

**DOI:** 10.1186/s13041-016-0192-8

**Published:** 2016-01-27

**Authors:** Federica Filice, Karl Jakob Vörckel, Ayse Özge Sungur, Markus Wöhr, Beat Schwaller

**Affiliations:** Anatomy, Department of Medicine, University of Fribourg, Route Albert-Gockel 1, CH-1700 Fribourg, Switzerland; Behavioral Neuroscience, Faculty of Psychology, Philipps-University of Marburg, Gutenbergstraβe 18, D-35032 Marburg, Germany

**Keywords:** Parvalbumin, Shank1, Shank3, Autism, Perineuronal net, Calcium-binding protein, Calcium homeostasis

## Abstract

**Background:**

A reduction of the number of parvalbumin (PV)-immunoreactive (PV^+^) GABAergic interneurons or a decrease in PV immunoreactivity was reported in several mouse models of autism spectrum disorders (ASD). This includes *Shank* mutant mice, with *SHANK* being one of the most important gene families mutated in human ASD. Similar findings were obtained in heterozygous (PV+/-) mice for the *Pvalb* gene, which display a robust ASD-like phenotype. Here, we addressed the question whether the observed reduction in PV immunoreactivity was the result of a decrease in PV expression levels and/or loss of the PV-expressing GABA interneuron subpopulation hereafter called “Pvalb neurons”. The two alternatives have important implications as they likely result in opposing effects on the excitation/inhibition balance, with decreased PV expression resulting in enhanced inhibition, but loss of the Pvalb neuron subpopulation in reduced inhibition.

**Methods:**

Stereology was used to determine the number of Pvalb neurons in ASD-associated brain regions including the medial prefrontal cortex, somatosensory cortex and striatum of PV-/-, PV+/-, *Shank1*-/- and *Shank3B*-/- mice. As a second marker for the identification of Pvalb neurons, we used *Vicia Villosa Agglutinin* (VVA), a lectin recognizing the specific extracellular matrix enwrapping Pvalb neurons. PV protein and *Pvalb* mRNA levels were determined quantitatively by Western blot analyses and qRT-PCR, respectively.

**Results:**

Our analyses of total cell numbers in different brain regions indicated that the observed “reduction of PV^+^ neurons” was in all cases, i.e., in PV+/-, *Shank1*-/- and *Shank3B*-/- mice, due to a reduction in *Pvalb* mRNA and PV protein, without any indication of neuronal cell decrease/loss of Pvalb neurons evidenced by the unaltered numbers of VVA^+^ neurons.

**Conclusions:**

Our findings suggest that the PV system might represent a convergent downstream endpoint for some forms of ASD, with the excitation/inhibition balance shifted towards enhanced inhibition due to the down-regulation of PV being a promising target for future pharmacological interventions. Testing whether approaches aimed at restoring normal PV protein expression levels and/or Pvalb neuron function might reverse ASD-relevant phenotypes in mice appears therefore warranted and may pave the way for novel therapeutic treatment strategies.

## Background

Autism spectrum disorders (ASD) consist of a group of heterogeneous neurodevelopmental disorders, with a high prevalence of ~1/100 children [1]. Core symptoms of ASD are deficits in social interaction and communication, together with restricted interests and repetitive behaviors [2–4]. The etiology of ASD remains unclear, but a strong genetic component is evident. ASD candidate genes are often implicated in synaptic transmission, are part of synapse formation/maintenance and/or affect the neurodevelopment during particular moments, e.g., during the “critical period” [5, 6]. Functionally, these changes affect the excitation/inhibition (E/I) balance and subsequently influence network properties [7, 8].

One of the most important gene families mutated in ASD is the *SHANK* gene family [9], coding for multi-domain scaffolding proteins located in the postsynaptic density of glutamatergic synapses [10]. In individuals with ASD or schizophrenia patients with ASD traits, mutations were repeatedly reported for all *SHANK* gene family members [4, 9, 11, 12], namely *SHANK1* [13], *SHANK2* [14–16] and *SHANK3* [17–21]. Moreover, *SHANK3* haploinsufficiency has been found in patients affected by the Phelan-McDermid 22q13 deletion mental retardation syndrome [22–25], often characterized by ASD features [26]. Importantly, mutations of *SHANK* genes were detected in the whole spectrum with a gradient in severity in mental retardation. Specifically, *SHANK1* mutations were found in individuals with ASD and normal intelligence, whereas *SHANK2* and *SHANK3* mutations were associated with mild and severe mental retardation, respectively [9]. Consistent with the important role of the *SHANK* gene family in ASD, genetic *Shank* mouse models display behavioral alterations with relevance to all human ASD core symptoms. *Shank1* null mutant mice display social and communication deficits [27], alterations in repetitive behavior, with elevated self-grooming behavior, particularly in social situations [28], and a mixed cognitive phenotype resembling aberrant cognitive processing evident in some ASD cases [29, 30]. Likewise in two *Shank2* models, strong ASD-related behavioral alterations are evident [17–19, 31, 32]. In the various *Shank3* models severity of the ASD phenotype varies with genetic manipulation, with a comparatively mild phenotype in the *Shank3* model lacking the ANK domain [33, 34], but strong phenotypes in the other models [35–37], see also [38–40].

In the process of brain development, GABAergic signaling plays an essential role. Thus, it is not surprising that its disturbance/disruption has been related to the pathogenesis of ASD [41, 42]. Investigations on the role of the GABAergic system during neurodevelopment, however, are impeded by the fact that GABAergic interneurons are made up of different subtypes displaying heterogeneous morphological and physiological features; in the hippocampus, up to 21 subtypes have been identified [43]. One way to classify GABAergic, e.g., cortical interneurons, is based on the expression of Ca^2+^-binding proteins such as parvalbumin (PV; gene symbol: *PVALB*), calbindin D-28k and calretinin [44]. Among these specific subpopulations, PV-expressing interneurons seem to be highly impacted in several neuropsychiatric disorders including schizophrenia, bipolar disorder and ASD [45]. While in none of the previous studies in humans the *PVALB* gene itself was found to be mutated, a reduction in the order of 20–25 % in the number of PV-immunoreactive (PV^+^) neurons has been reported in ASD individuals [46] and mouse ASD models [47], see Table S1 in [48]. However, so far the question was not thoroughly addressed as to what extent the reduction in PV^+^ neurons was the result of improper neurodevelopment (e.g., altered GABA interneuron subtype), neuron loss (neuronal death) or PV down-regulation (mRNA and/or protein). This is of high relevance, since the alternatives likely have distinct and even opposing effects on the excitation/inhibition balance: while loss of the Pvalb neuron subpopulation is likely to result in reduced inhibition, decreased PV expression results in enhanced inhibition. The absence of PV in PV-/- Pvalb neurons does not affect basal synaptic transmission, but enhances facilitation [49, 50] and shortens delayed transmitter release [51]. This asynchronous release augmented in the presence of PV is assumed to be important to desynchronize large fractions of local networks and prevent/disrupt excessive synchronized activity [52]. In line the observed increased regularity of spiking of PV-/- striatal Pvalb FSI in vitro [53], the appearance of synchronous 160-Hz oscillations in the cerebellum of PV-/- mice in vivo [54] and facilitation of the GABA_A_-ergic current reversal caused by high-frequency stimulation in PV-/- hippocampal Pvalb FSI in vitro [55], all together provide evidence that PV plays a key role in the regulation of local inhibitory effects on pyramidal neurons, as well as on other interneurons (for more details, see [56]). Importantly, at the behavioral level, mice with reduced PV expression (PV+/-) or without PV (PV-/-) display a robust ASD-like phenotype [48]. Although qualitative immunohistochemistry revealed no striking differences with respect to VVA^+^ (putatively Pvalb) neurons in PV-/- mice [55, 57], a quantitative and systematic analysis of Pvalb neurons by unbiased stereology in different brain regions including ASD-implicated regions such as the medial prefrontal cortex (mPFC), somatosensory cortex (SSC) and striatum was still missing. Besides determining the number of Pvalb neurons in PV+/- and PV-/- mice, we quantified the number of this interneuron subpopulation in two well-established ASD mouse models, i.e., *Shank1*-/- and *Shank3B*-/- mice, covering the extremes of the spectrum with a gradient in severity in mental retardation.

## Methods

### Animals

All mice were group housed in temperature-controlled animal facilities (24 °C, 12:12 h light/dark cycle), either at the University of Fribourg, Switzerland, or at the University of Marburg, Germany, and fed ad libitum. PV-deficient (PV-/-; strain name: B6.Pvalb^tm1Swal^) mice were generated by homologous recombination as previously described [58] and are considered as congenic with C57Bl/6 J [59]. C57Bl/6 J wild-type (WT) mice were used to generate the heterozygous PV+/- group. In some experiments mice expressing EGFP in the Pvalb neurons (line B6.Tg(Pvalb-EGFP)1Hmon [53]) were used. *Shank1*-/- mice (B6.129S4-Shank1^tm1Shng^/J) were generated in the laboratory of M. Sheng (29). *Shank3B*-/- mutant mice (B6.129-Shank3^tm2Gfng^/J; http://jaxmice.jax.org/strain/017688.html) were purchased from The Jackson Laboratory (Bar Harbor, Maine, USA) and were initially generated by Peca and colleagues [36]. Genotypes were determined according to established and previously described protocols [28, 36, 58]. Only male animals were used in this study. All experiments were performed with permission of the local animal care committees (Canton of Fribourg, Switzerland; Regierungspräsidium Gießen, Germany) and according to the present Swiss/Germany law and the European Communities Council Directive of 24 November 1986 (86/609/EEC).

### Tissue preparation and immunohistochemistry

Mice at postnatal day 25 (PND25) were anesthetized (Esconarkon, Streuli Pharma AG, Uznach, Switzerland) and perfused with 0.9 % saline solution followed by 4 % PFA. Brains were removed and post-fixed for 24 h in 4 % PFA before being cryopreserved in 30 % sucrose-TBS at 4 °C. Coronal and sagittal sections were cut rostro-caudally using a freezing microtome (Frigomobil, Reichert-Jung, Vienna, Austria) and six series of equidistant sections were collected using stereological systematic random sampling principles (see below). Free-floating sections were first incubated with TBS 0.1 M plus 0.4 % Triton X-100 and 10 % newborn calf serum (NBS) for 1 h at room temperature, then washed three times with TBS 0.1 M, and incubated with PV antibody (anti-rabbit PV25, Swant, Marly, Switzerland) at a dilution 1:1000 and *Vicia Villosa Agglutinin* (biotinylated-VVA, Reactolab, Servion, Switzerland); 10 μg/ml in TBS 0.1 M plus MgCl_2_, MnCl_2_, CaCl_2_ (final salt concentration: 0.1 mM) overnight at 4 °C. Sections were washed once with TBS, then twice with Tris-HCl and incubated protected from light at room temperature with anti-rabbit Cy3-conjugated antibody (1:200 dilution) and Alexa488 streptavidin-conjugated antibody (1:200 dilution, Milan Analytic AG, Switzerland) in Tris-HCl. Nuclei of fixed cells were stained with DAPI (1:500 dilution, LuBio Science GmbH, Luzern, Switzerland) in PBS 0.1 M. After rinsing, slides were coverslipped with Hydromount (National Diagnostics, Atlanta, Georgia, USA).

### Stereological quantification

The optical fractionator method [60] was used to estimate the total number of parvalbumin-positive (PV^+^) and Vicia Villosa Agglutinin-binding (VVA^+^) cells in brain regions of interest (ROIs) using the Stereo Investigator system (Version 11, MicroBrightField, Williston, VT, USA). The system was connected to a Zeiss Axioplan microscope coupled with a Hamamtsu Orca Camera and with a motorized x-y stage (Ludl Electronic Products, LTD, NY, USA). ROIs were determined based on stereotactic coordinates provided by the Paxinos and Franklin atlas [61] at 1.78–1.18 from bregma for the medial prefrontal cortex (mPFC), 1.10 to−0.46 mm from bregma for the striatum and 1.20–3.25 mm lateral to the midline for the somatosensory cortex (SSC). Counting was done on images obtained with oil immersion objective lenses (x100 NA = 1.40 and x63 NA = 1.30). Five animals derived from heterozygous breedings were analyzed per genotype, with mice from the same litter but different genotypes, i.e., littermate controls, being included.

### Counting criteria

**Fig. 1:**
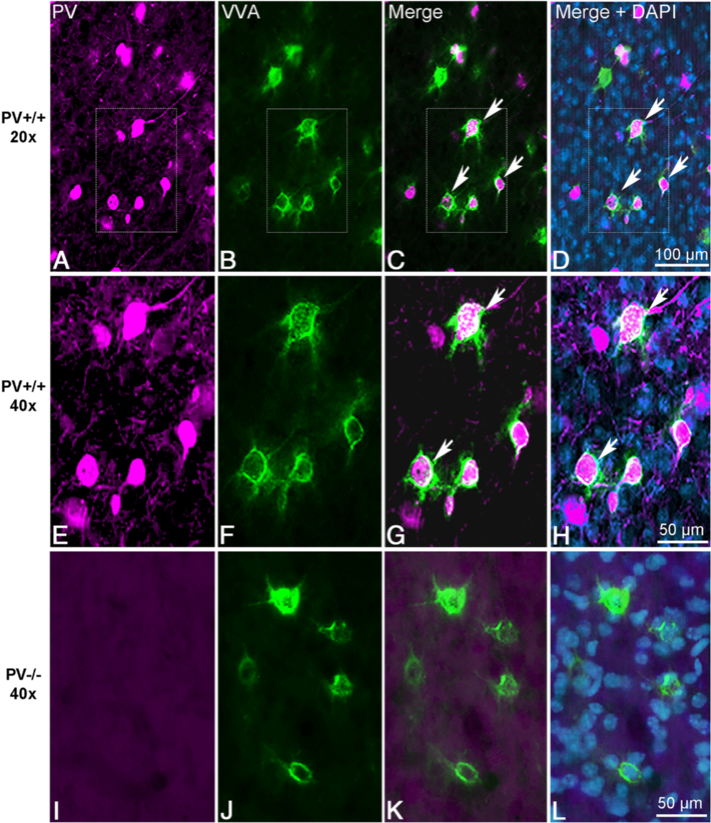
Representative PV^+^ and VVA^+^ cells from PND25 mouse cortex. **a**, **b** Single channel acquisition of (**a**) PV^+^ (*magenta*) and (**b**) VVA^+^ (*green*) cells. (**c**, **d**) Merged images showing PV (*magenta*) and VVA (*green*) overlapping in the PND25 cortex of a WT mouse, in (**d**) additionally with DAPI (*light blue*) counterstaining. In PV^+^ cell bodies (*magenta*), where most of the cell including the nucleus evidenced by DAPI staining (*blue*) was within the thickness of the section, the PNN (*green*) surrounding the cell was clearly visible. **a**–**d) ** Low magnification and **e**–**h**) High magnification images. Arrowheads indicate the PV^+^VVA^+^ double-positive neurons that are in focus. In the overlay image, the rim of the cells was lighting up in white, indicative for “co-localization” of PV and VVA. **i**–**l**) High magnification images of PV^+^ (*magenta*) and VVA^+^ (*green*) cells from PND25 PV-/- mouse cortex. The morphology of PNNs in PV-/- animals does not differ from the one of WT mice. Scale bar: 100 μm (**a**–**d**), 50 μm (**e**–**l**)

Sampling parameters are reported in Table 1. VVA^+^ and PV^+^ cells were counted independently and according to the following criteria: (1) Well visible DAPI-stained nucleus; (2) well-defined perineuronal net (PNN) with a web-/lattice-like morphology for VVA^+^ cells; examples are shown in Fig. 1) and (3) PV staining surrounding the DAPI-stained nucleus for PV^+^ cells. Section thickness was measured at every fifth sampling location, and the mean of all measurements was used in all computations. At each sampling location, the microscope was focused down through the disector sample to count any cell within that particular counting frame according to disector counting rules. Since the fractionator method does not require a measurement of tissue volume or any other dimensional quality, the cell number estimate is valid, even if the tissue volume changes during processing. The total number of cells (N) in the ROIs was estimated as outlined by West et al. [60, 62] using the equation:

**Table 1:** Stereological sampling parameters

Brain region	Cutting plane	No. of sections	Section evaluation interval	Height of disector (μm)	Guard zone height (μm)	Counting frame area (μm)	Sampling grid area (μm)	Measured section thickness mean (μm)	CE
mPFC	coronal	4-5	6	20	0.5	90 × 70	150 × 200	22.4	CE_m=1_0.07 ≤ CE ≤ CE_m=0_ 0.09
Striatum	coronal	7-8	6	20	0.5	110 × 90	300 × 300	23.0	CE_m=1_0.06 ≤ CE ≤ CE_m=0_0.09
SSC	sagittal	10-12	6	20	0.5	583.5 × 335.6	250 × 250	23.4	CE_m=1_0.07 ≤ CE ≤ CE_m=0_0.10

$$ \mathrm{N} = \sum {\mathrm{Q}}^{-}\mathrm{x}\ \left(1/\mathrm{s}\mathrm{s}\mathrm{f}\right)\ \mathrm{x}\ \left(1/\mathrm{a}\mathrm{s}\mathrm{f}\right)\ \mathrm{x}\ \left(1/\mathrm{t}\mathrm{s}\mathrm{f}\right), $$

where ssf, asf and tsf are referred to the sampling sample fraction, the area sampling fraction and the thickness sampling fraction, respectively. The precision of the estimates N is described by the coefficient of error CE (Table 1), which is the sampling error related to counting noise, systematic uniform random sampling and variances in section thickness [63]. A CE of 0.10 is generally accepted for most biological samples. CEs for both *m* = 1 and *m* = 0 are provided and are likely to bracket the true CEs of the estimates. Unlike in most double-labeling studies, strictly individual images for either PV^+^ or VVA^+^ structures were counted without crosschecking the other channel. In such a way, a cell, or more likely a cell segment resulting from the sectioning with “low” staining intensities (below the predefined threshold) and/or “atypical” shape (i.e., not easily discernable as cell-like) was considered negative, even if a check of the other channel would have identified this cell or cell segment as (likely also weakly) positive for the second marker.

### RT-qPCR

Total RNA was extracted from mouse brain tissue using peqGold TRIzol reagent (Peqlab, VWR International GmbH, Erlangen, Germany). cDNA was synthesized using Promega’s reverse transcriptase kit (Promega AG, Dübendorf, Switzerland). qRT-PCR was carried out to examine the expression of mRNA of the *18S rRNA* and *Pvalb* genes using the universal 2X KAPA SYBR FAST qPCR Master Mix (Axonlab AG, Mont-sur-Lausanne, Switzerland). Details about the primer sequences are listed in Table 2. Gene expression quantitation was carried out in a DNA thermal cycler (Corbett Rotor gene 6000, QIAGEN Instruments AG, Hombrechtikon, Switzerland), according to the following two-steps protocol: a denaturation step of 95 °C for 3 min; 40 cycles of denaturation at 95 °C for 3 s and annealing/extension/data acquisition ranging from 60 to 62 °C for 20 s. The housekeeping gene 18S ribosomal RNA (*18S*) was used as an endogenous control to normalize the mRNA content for each sample. Normalized mRNA levels were quantified by the 2 − △△Ct method [64].

**Table 2:** qRT-PCR primers

Primer	Sequence 5’-3’	Nt position	Gene	Gene accession number
18S rRNA	For: TCAAGAACGAAAGTCGGAGGTT	1026–1047	*Rn18s*	NR_003278
	Rev: GGACATCTAAGGGCATCACAG	1493–1513		
PV	For: TGTCGATGACAGACGTGCTC	24–43	*Pvalb*	NM_013645
	Rev: TTCTTCAACCCCAATCTTGC	309–328		

### Western blot analyses

Brains from euthanized mice were quickly removed, homogenized and soluble proteins extracted for Western blotting experiments as described before [65]. Proteins (30 μg) were separated by SDS-PAGE (15 %). After electrophoresis, the proteins were transferred on nitrocellulose membranes (MS solution, Chemie Brunschwig, Basel, Switzerland). The membranes were then blocked in 5 % non-fat milk in TBS-T for 60 min at room temperature and incubated with primary antibodies: rabbit anti PV25 (Swant, Marly, Switzerland), rabbit anti Calbindin D-28k (Swant, Marly, Switzerland) diluted 1:10,000 in 2 % non-fat milk in TBS-T overnight at 4 °C. Membranes were washed three times in TBS-T and incubated for 1 h with secondary antibody (goat anti-rabbit IgG HRP conjugated, Sigma-Aldrich, Buchs, Switzerland) diluted at 1:10,000 in TBS-T. Finally, membranes were repeatedly rinsed in TBS-T and developed using ECL (Merck Millipore, Schaffhausen, Switzerland). Bands visualized by ECL were quantified using Alpha VIEW SA software (California, USA). The levels of PV signals were normalized to calbindin D-28k (CB) signals, a protein previously shown to be unaltered in PV-/- mice [57, 66] to control for differences in loading. CB levels were found to be unaltered in *Shank1*-/- and *Shank3B*-/- brains (data not shown). Alternatively the GAPDH signal on Western blots or the integral of the protein signals per sample of the Ponceau Red-stained membranes was used for normalization. No significant differences existed between PV signals quantitatively evaluated by either normalization method (not shown).

### Statistical analysis and cell number estimates

mRNA and protein levels were compared between genotypes by the Student's *t*-test. Stereological data were analyzed using the GraphPad Prism software (San Diego, USA). Since no differences were encountered when comparing the ROIs of the two hemispheres in the same animal, data were pooled together and analyzed. The morphological data were first checked for normal distribution by the Kolmogorov-Smirnov test and then analyzed with a one-way ANOVA with genotype as factor. Tukey’s test was performed as *post hoc* test. A *p*-value <0.05 was considered statistically significant.

## Results

### The numbers of PV^+^ (Pvalb) interneurons are not changed in PV-reduced (PV+/-) and in PV-deficient (PV-/-) mice

Parvalbumin fast-spiking interneurons (PV-FSI) are preferentially ensheathed by perineuronal nets (PNNs) [67, 68] consisting of specialized extracellular matrix components that form a lattice-like structure around the somata and proximal dendrites of PV-expressing neurons. Various staining methods allow visualizing PNNs including *Vicia Villosa Agglutinin* (VVA), a lectin that binds to N-acetyl-galactosamine residues of PNN components. PV^+^ and VVA^+^ cells were identified by fluorescent staining methods. Since neither the morphology nor the distribution of PNNs were shown to be altered in PV+/- and PV-/- mice in comparison to PV+/+ mice [57, 69], VVA staining was used as reliable marker for visualizing the specific neuronal subpopulation of “PV-FSI” cells, defined as Pvalb neurons, irrespective of reduced or absent PV expression in PV+/- and PV-/- mice, respectively. Representative samples demonstrating the quality of the stainings for PV and VVA of PND25 PV+/+ and PV-/- mouse brains are depicted in Fig. 1. As expected and reported before [57], no positive staining for PV was detectable in the sections from PV-/- mice (Fig. 1).

**Fig. 2:**
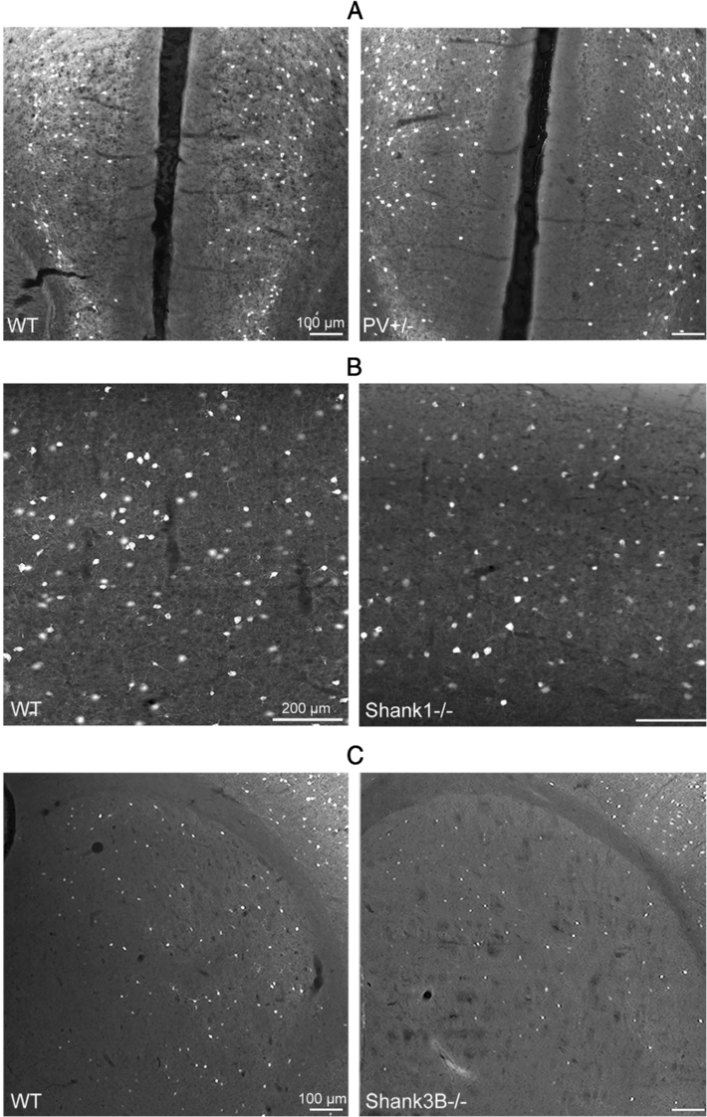
PV immunofluorescence images from mPFC of a PV+/- (**a**), SSC of a *Shank1*-/- (**b**) and striatum of a *Shank3B*-/- (**c**) mouse in comparison to the same regions of a WT mouse. PV expression levels (≈ signal intensity) varied considerably between individual PV^+^ neurons. Note the generally weaker somatic staining in the mutant mice (right panels), also evident by the fainter staining of the PV-ir neuropil. The weaker staining results in a lower number of neurons considered as positive for PV as shown in Figs. 3, 4 & 6. Scale bars: 100 μm in **a**, **c**; 200 μm in **b**

**Fig. 3:**
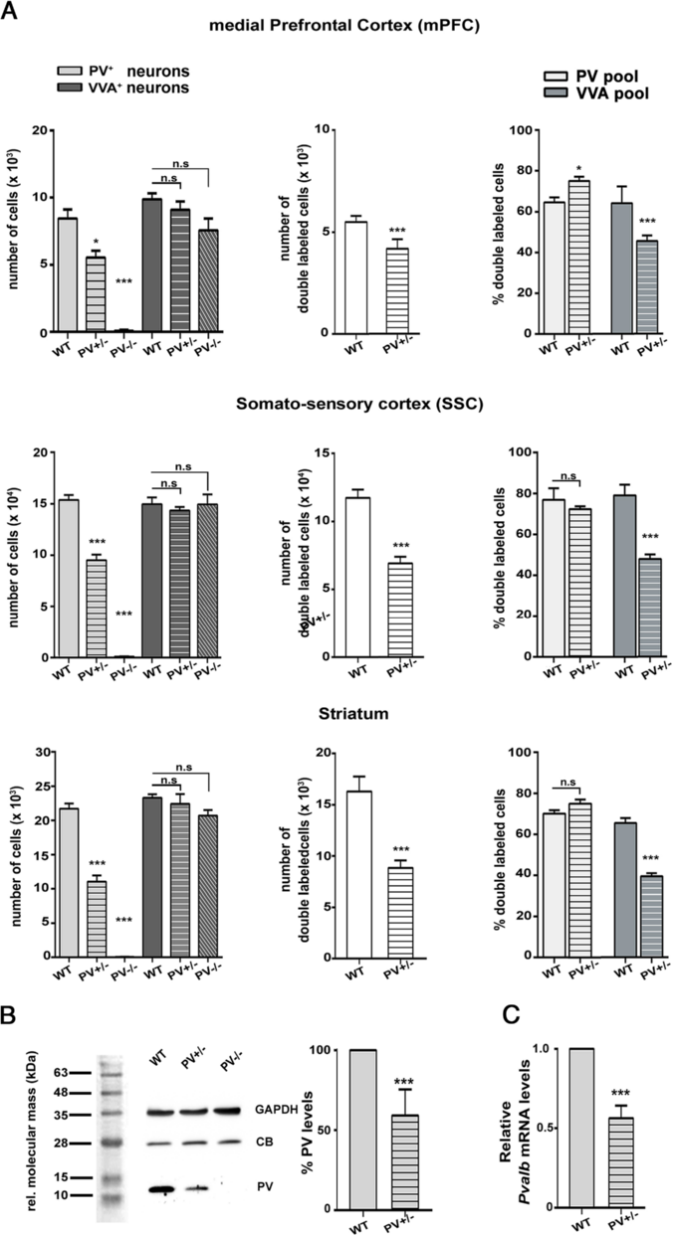
**a** *Left*: Stereological estimations of PV^+^ (*light gray*) and VVA^+^ (*dark gray*) cells in mPFC (*upper row*), SSC (*middle row*) and striatum (*lower row*) of PND25 WT, PV+/- and PV-/- male mice. Significant differences are observed in PV^+^ cells between WT, PV+/- and PV-/- animals (*p*-value <0.05). *Significant vs. WT mice. Asterisks represent *P ≤ 0.05, **P ≤ 0.01, ***P ≤ 0.001, respectively. Middle: stereological estimation of double-labeled (PV^+^ VVA^+^) cells of PND25 WT and PV+/- mice (white bars); Right: percentage of PV^+^ cells surrounded by VVA (*light gray*) and VVA^+^ cells showing PV expression (*dark gray*) in WT and PV+/- mice. **b** Quantitative Western blot analysis of forebrain samples of P25 WT PV+/- and PV-/- mice. A representative Western blot (*left*) and the quantification of PV protein levels in WT and PV+/- forebrain are shown (*right*). No PV signal was detectable in PV-/- mice. The Ponceau Red-stained protein markers loaded on the same membrane as the brain extract samples are shown with their respective molecular mass on the left. Data are from three independent experiments and are shown as mean ± SEM. Results are expressed as a percentage of normalized PV levels measured in control (WT), defined as 100 %. GAPDH or calbindin D-28k (CB) signals served as loading controls and were used for the normalization of the PV signals. Both, CB and GAPDH expression levels were unchanged in PV+/- and *Shank* mutants compared to WT mice (data not shown). **c** qRT-PCR values from P25 PV+/- mice representing *Pvalb* mRNA levels were normalized to 18S mRNA levels and expressed as fold change compared to WT. Data from three independent experiments were pooled together and are shown as mean ± SD. In all graphs, asterisks indicate statistical significance vs. WT (p-value <0.05, *p* = 0.0003)

Cell numbers were estimated using the optical fractionator method in three selected ROIs: the medial prefrontal cortex (mPFC), the somatosensory cortex (SSC) and the striatum of PV+/+, PV+/- and PV-/- mice. CE values ranged from 0.06 to 0.10 (Table 1) indicating a sufficient precision of the estimates for both cell populations [63]. We observed a significant reduction in the number of PV^+^ interneurons in PV+/- and PV-/- mice compared to PV+/+ in all ROIs. On average, PV^+^ cells in PV+/- mice were reduced to 60 % of PV+/+ controls; representative immunofluorescence images of mPFC from a WT and a PV+/- mouse are shown in Fig. 2a. The reduction in PV staining intensity in PV+/- samples is most striking by the decreased intensity of neuropil staining as previously observed in the temporal cortex (Fig. 2a in [57]). Evidently, the count in PV-/- cells was essentially zero. With respect to the number of VVA^+^ cells, no significant differences existed between all three genotypes and all three ROIs (Fig. 3a, left). An average of the three regions resulted in values of 98 % and 99 % of WT control for PV+/- and PV-/-, respectively, indicating that while PV expression levels were decreased, the number of Pvalb cells was unchanged (Table 3). Since expression of PV and PNNs are developmentally regulated, modulated by neuronal activity, e.g., during “sensitive” or “critical” periods, and moreover altered under pathological conditions (see discussion), we additionally used a transgenic mouse line, where EGFP is expressed under the control of the *Pvalb* promoter [53, 70] to estimate the number of Pvalb cells. Cell counts of EGFP^+^ cells in PV+/+EGFP mice revealed the number of positive cells to be nearly identical to either PV^+^ or VVA^+^ cell numbers in the three ROIs, i.e., mPFC, SSC and striatum (Table 3). This indicates that the number of VVA^+^ cells represents a reliable estimate of the Pvalb cells.

**Table 3:** Mean total number of PV^+^ and VVA^+^ cells in the mPFC, SSC and striatum of PV+/+, PV+/-, PV-/-, as well as in PV+/+EGFP reporter mice

	mPFC PV^+^			mPFC VVA^+^		
	Mean	Range	*p*-value	Mean	Range	*p*-value
PV+/+	8908	7606–10,417		9292	6921–10,987	
PV+/-	5541	4321–7194	0.0003	9015	7901–10,627	0.3429
PV-/-	--	--	<0.0001	7421	5343–10,589	0.0503
PV+/+ EGFP	8732	6915–9713	0.7861	8664	7202–10,093	0.1079
	SSC PV^+^			SSC VVA^+^		
	Mean	Range	*p*-value	Mean	Range	*p*-value
PV+/+	153729	144,549–166,441		149709	129,498–169,086	
PV+/-	95157	82,476–109,745	<0.0001	143609	137,087–154,975	0.4419
PV-/-	--	--	<0.0001	149594	120,020–174,596	0.9925
	striatum PV^+^			striatum VVA^+^		
	Mean	Range	*p*-value	Mean	Range	*p*-value
PV+/+	23332	22,384–25,012		21724	20,003–24,431	
PV+/-	11036	8330–13,501	<0.0001	22445	17,158–24,801	0.6613
PV-/-	---	---	<0.0001	20713	17,682–22,348	0.3875
PV+/+ EGFP	18562	17,670–19,681	<0.0001	19881	18,158–21,118	0.0875

We then determined the number of cells positive for both PV and VVA in PV+/+ and PV+/- mice. The percentage of double-labeled cells was rather homogeneous among brain regions and in the order of 70 % in PV+/+ and 55 % in PV+/- brains (Fig. 3a, middle). The difference between genotypes was significant in all ROIs. For this calculation in PV+/+ mice, we used the average of the PV^+^ and VVA^+^ cells, reckoning that this number represented the “real” number of Pvalb neurons; the use of PV^+^ cell number only would not have significantly changed the results presented in Fig. 3a (not shown). Although PNNs enwrap mostly PV^+^ neurons [69, 71], this overlap is not absolute. Hence, we first calculated the percentage of PV^+^ cells that are surrounded by VVA^+^ PNNs. This percentage was found to be ~75 % in all the ROIs and there were no significant differences between PV+/+ and PV+/- mice (Fig. 3a, right). Vice versa, VVA^+^ cells that also showed PV staining accounted for around 68 % in PV+/+ mice for all the three ROIs. Values were clearly lower in PV+/- mice, on average 45 %, i.e., approximately one third fewer double-labeled cells compared to WT (Fig. 3a, right). This indicates that all identified PV^+^ cells were highly likely to show VVA staining, while in a fraction of VVA^+^ cells, PV expression levels, more prominently in PV+/- mice were below the predefined PV expression threshold and were thus not considered as double-labeled cells. PV protein levels in the forebrain of PND25 PV+/- mice were reduced by approximately 50 % determined by semi-quantitative Western blot analyses (Fig. 3b), in line with previous findings in PV+/- adult mice [57]. No Western blot signal for PV was observed in samples from PV-/- mice as shown before [49, 57]. For the normalization of the PV signals either CB or GAPDH bands were used (Fig. 3b) and results were not different when using either protein for the normalization. *Pvalb* mRNA levels were decreased to a similar extent (≈50 %) indicative of a regulation at the transcriptional level (Fig. 3c).

### PV expression levels are reduced in two canonical ASD models, *Shank1*-/- and *Shank3B*-/-, while VVA^+^ Pvalb neuron numbers are unaltered

As already reported in several studies [47, 72–74] canonical ASD mouse models were shown to display a reduction in the number of PV-immunoreactive cells in various brain regions. In most studies this was assumed to result from a cell loss of the PV^+^ subpopulation. Although ASD-linked gene defects/mutations were found to be of rather non-homogeneous origin, including proteins implicated in synaptic transmission, neurodevelopment, transcriptional regulation, etc., the morphological brain alterations observed in 26 ASD mouse models allowed a grouping into 3 major subgroups [75]. Knockout mice in group 1 including null mutants for *En2*, *Fmr1* and *Shank3* show an increase in the frontal and parieto-temporal lobes and a volume decrease in the cerebellum, as well as a decrease in PV staining. Similar neuroanatomical changes are present in PV-/- (*Pvalb*) mice, i.e., a transient cortical hypertrophy and a decrease in cerebellar volume at young age [48], indicating that PV-/- likely belongs to the group 1 ASD models. Thus we extended our studies to two well-established genetic mouse models for ASD, namely *Shank1*-/- and *Shank3B*-/- mice. The two models display behavioral phenotypes with relevance to all core symptoms of ASD in humans [38–40] and cover the extremes of the spectrum with a gradient in severity in mental retardation [9]. Global PV expression levels as well as the ones in PV^+^ neurons in various brain regions are currently unknown in these two mouse mutants; a decrease in PV staining intensity and puncta density of PV-ir neurons contacting pyramidal cells in the mouse insular cortex has been reported in *Shank3B*-/- mice [76].

Since expression patterns of the two proteins are quite different, i.e., high mRNA levels of *Shank1* in cortex and hippocampus and of *Shank3* in striatum and cortex [36, 77] (see also mouse Allen Brain atlas), we hypothesized to find the largest differences in PV expression in regions with high expression of the two Shank members. Thus, SSC and striatum were analyzed for *Shank1*-/- and *Shank3B*-/- mice, respectively. The number of PV^+^ and VVA^+^ cells was estimated as for the PV null-mutant mice by the optical fractionator method using the same counting criteria.

**Fig. 4:**
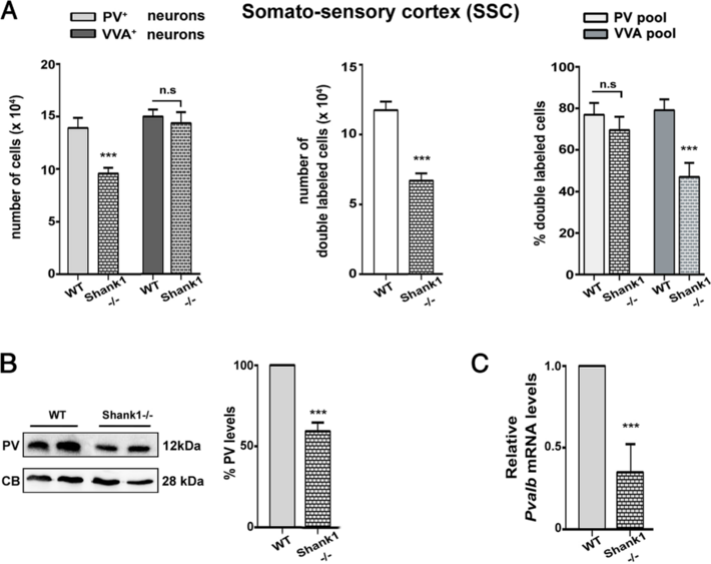
**a** *Left*: stereological estimations of PV^+^ (*light gray*) and VVA^+^ (*dark gray*) cells in SSC of WT and *Shank1*-/- PND25 male mice. *Middle*: estimation of double-labeled cells in SSC of PND25 WT and *Shank1*-/- mice. Right: percentage of PV^+^ cells surrounded by VVA (*light gray*) and percentage of VVA^+^ cells displaying PV expression and thus identified as PV^+^ cells (*dark grey*) in WT and *Shank1*-/- mice. **b** Representative Western blot and quantification of PV protein levels in *Shank1*-/- mice. CB signals were used as loading controls and served for the normalization of the PV signals. Data are from three independent experiments and are shown as mean ± SEM. Results are expressed as percentage of normalized PV levels measured in control (WT) samples, defined as 100 %. **c** Quantitative RT-PCR analysis of forebrain samples of *Shank1*-/- mice. PV (*Pvalb*) mRNA levels were normalized to 18S mRNA levels and expressed as fold change. Data from three independent experiments were pooled together and are shown as mean ± SD. In all graphs, asterisks indicate statistical significance vs. WT (*p*-value <0.05, *p* = 0.0006)

**Fig. 5:**
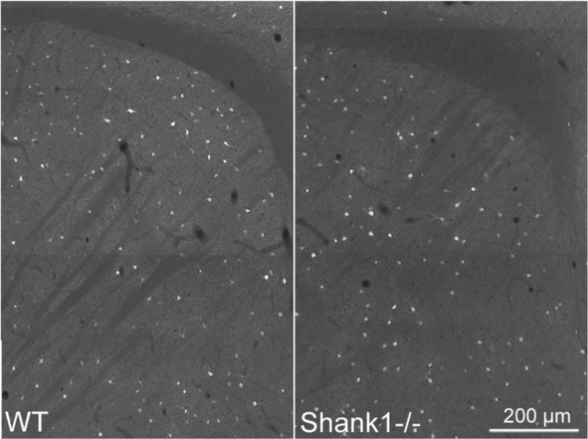
Representative PV immunofluorescence images from the striatum of a WT and a *Shank1*-/- mouse. No qualitative differences in the number and signal intensities of PV^+^ neurons were observed between genotypes. Scale bar: 200 μm

Results in the SSC of *Shank1*-/- mice were highly similar to the ones observed in the SSC of PV+/- mice. The number of PV^+^ cells was significantly reduced to 62 % of PV+/+ in *Shank1*-/- mice, while the number of VVA^+^ cells was unaltered (Fig. 4a, left & Table 4); representative PV immunofluorescence images of SSC from a WT and a *Shank1*-/- mouse are depicted in Fig. 2b. The lower PV levels not only led to weaker somatic PV signals, but also the staining intensity of the PV-positive neuropil was clearly reduced. Also results from the co-localization experiments (Fig. 4a, middle & right) are essentially identical to the ones of PV+/- strongly indicative of PV down-regulation and not PV^+^ cell loss. In support, quantitative RT-PCR results and moreover Western blots showed that both PV mRNA level and protein levels, respectively (Fig. 4b, c), were significantly decreased, demonstrating that the PV down-regulation occurs at the transcriptional level. As we expected to detect the largest differences in PV expression levels in brain regions with high *Shank1* expression, as a control we also investigated PV expression in the striatum, a region with low *Shank1* expression levels evidenced by ISH [36]. Qualitatively, immunofluorescence images revealed no prominent differences with respect to staining intensity and PV^+^ neuron density between WT and *Shank1*-/- mice in the striatum (Fig. 5).

**Table 4:** Mean total number of PV^+^ and VVA^+^ cells in the SSC of *Shank1*-/- and in the striatum of *Shank3B*-/- mice in comparison to WT (PV+/+) mice in the two ROIs

	SSC PV^+^			SSC VVA^+^		
	Mean	Range	*p*-value	Mean	Range	*p*-value
PV+/+	153729	144,549–166,441		149709	129,498–169,086	
Shank1-/-	96000	85,803–114,459	<0.0001	143392	128,601–166,705	0.6250
	striatum PV^+^			Striatum VVA^+^		
	Mean	Range	*p*-value	Mean	Range	*p*-value
PV+/+	23332	22,384–25,012		21724	20,003–24,431	
Shank3B-/-	12881	10,132–14,808	<0.0001	19951	16,887–22,811	0.1910

**Fig. 6:**
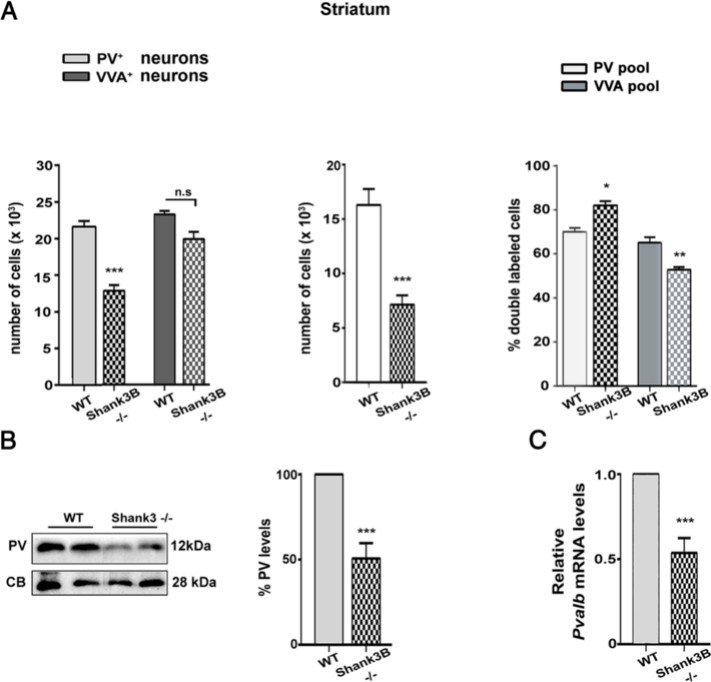
**a**) *Left*: stereological estimations of PV^+^ (*light gray*) and VVA^+^ (*dark gray*) cells in the striatum of WT and *Shank3B*-/- PND25 male mice. Middle: estimation of double-labeled cells in SSC of PND25 WT and *Shank3B*-/- mice. Right: percentage of PV^+^ cells surrounded by VVA (*light gray*) and VVA^+^ cells with PV expression (*dark grey*) in WT and *Shank3B*-/- mice. **b**) Representative Western blot and quantification of PV protein levels in *Shank3B*-/- mice. CB was used as loading control for the normalization of the PV signal, since CB expression levels were unchanged in *Shank3B*-/- mice (data not shown). Data are from three independent experiments and are shown as mean ± SEM. Results are expressed as a percentage of normalized PV levels measured in control (WT), defined as 100 %. **c**) Quantitative RT-PCR analysis of forebrain samples of *Shank3B*-/- mice. PV (*Pvalb*) mRNA levels were normalized to 18S mRNA levels and expressed as fold change. Data from three independent experiments were pooled together and are shown as mean ± SD. In all graphs, asterisks indicate statistical significance vs. WT (p-value <0.05, *p* = 0.0002)

**Fig. 7:**
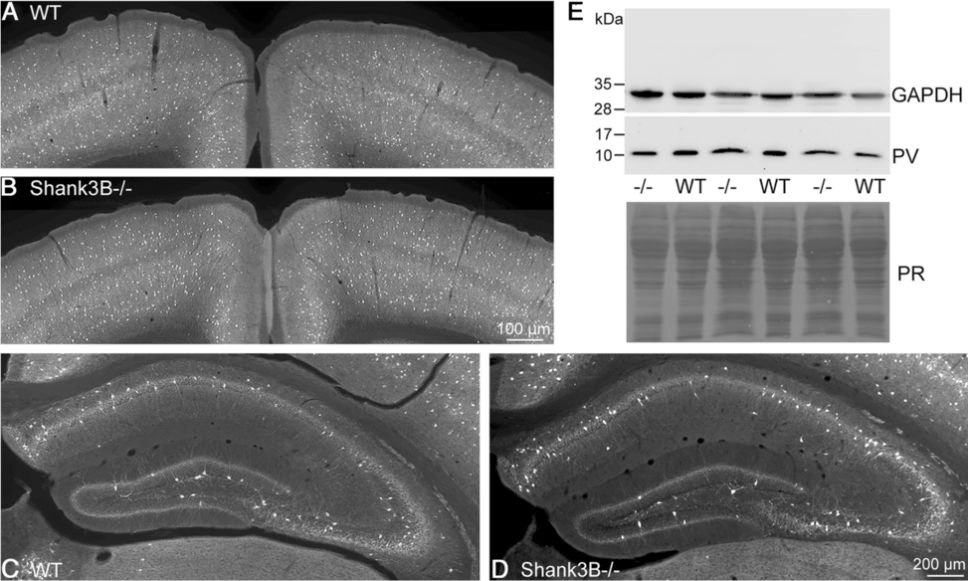
Representative PV immunofluorescence images (**a**–**d**) and Western blot analyses (**e**) from cortex and hippocampus of WT (**a**, **c**) and *Shank3B*-/- (**b**, **d**) mice. No apparent differences in the number or signal intensity of PV^+^ neurons in both brain regions were evident between WT and *Shank3B*-/- mice. Scale bars in **a** and **b**: 100 μm; in **c** and **d**: 200 μm. **e**) Western blot signals from brain homogenates containing cortex and hippocampus showed relatively high signal variability independent of the genotype. For the normalization of the PV signal either the GAPDH Western blot signal (upper part) or the intensity of the Ponceau Red-stained membrane (PR) was used. Results from 3 WT and 3 *Shank3B*-/-mice are depicted. The positions of the molecular weight markers are indicated. Due to the large difference in signal intensities for GAPDH and PV, the membrane was cut and exposed individually

For the *Shank3B*-/- mice we focused on the striatum, a region with high *Shank3* expression levels, as well as containing the subpopulation of PV-FSI, whose function were previously shown to be altered in the absence of PV [53]. While the number of VVA^+^ cells was unchanged, the number of PV^+^ cells was reduced to approximately 58 % (Fig. 6a, left & Table 4). The decrease was clearly visible on immunofluorescence images of the striatum of a *Shank3B*-/- mouse in comparison to a WT animal (Fig. 2c). The co-localization studies also revealed a decrease of double-labeled cells, again mostly resulting from a reduction in PV^+^ cells showing VVA staining (Fig. 6a, middle). Interestingly, in the *Shank3B*-/- mice, the number of PV^+^ cells also expressing VVA was even slightly increased (Fig. 6a, right). However, counting and co-localization results show globally similar results as for PV+/- and *Shank1*-/- mice, i.e., a reduction of PV expression in the unchanged number of striatal VVA^+^ cells. Both RT-PCR and Western blot analysis revealed the PV mRNA and protein expression levels to be significantly lower, i.e., to 53 ± 8 and 50 ± 9 % of PV+/+ levels for mRNA and protein, respectively (Fig. 7b, c). Based on the rather low *Shank3* ISH signals in the cortex and the hippocampus, we hypothesized to observe minor (if any) effects on PV expression in these brain regions. Qualitatively, immunofluorescence images of cortex (Fig. 7a, b) and hippocampus (Fig. 7c, d) revealed no obvious differences between WT (a, c) and *Shank3B*-/- (b, d) mice. Quantitatively, the PV Western blot signals (Fig. 7e) were not significantly different between genotypes (100 ± 15 % in WT vs. 103 ± 25 %; in *Shank3B*-/-; mean ± S.D., *n* = 5 mice per genotype; *p* = 0.78).

Thus, in both *Shank* knockout ASD models, PV protein expression levels were decreased in those brain regions normally expressing high levels of either *Shank1* or *Shank3* in WT mice likely arising from alterations at the transcriptional level, in view of the similar decrease in *Pvalb* mRNA. Importantly, in all three ASD models there was no evidence for a Pvalb neuron loss.

## Discussion

One of the difficulties in gaining knowledge on the mechanisms underlying the pathogenesis of ASD is the large number (>100) of putative ASD risk genes identified in genetic studies in humans and animal models [4, 78, 79]. An important line of evidence has centered on mutations in genes implicated in synapse structure and/or function including *NRXN*, *NLGN* and *SHANK* family members [17–19, 80]. Such mutations might eventually lead to an alteration of the E/I balance as demonstrated in some ASD mouse models [7, 8]. Other hypotheses on ASD-associated genes and/or gene/environment relationships come from many computational studies (GWAS, transcriptomic expression network analyses); several ones revealed impaired Ca^2+^ signaling, i.e., alterations in the Ca^2+^ node in gene networks [4, 81, 82] to represent a convergence of mechanisms relating to ASD, in line with previous propositions [83–85]. PV plays an important role in the Ca^2+^ homeostasis regulating many aspects of neuronal signaling (short-term plasticity, synchronization, precision of spike timing, etc.) in the subpopulation of PV^+^ interneurons [56]. Absence/down-regulation of PV in PV-/- and PV+/- mice, respectively, not only affects the properties of the Pvalb neurons, but also of neurons impacting on Pvalb cells, and leads to a robust ASD-like behavioral phenotype characterized by impaired social interaction behavior, reduced pro-social ultrasonic vocalizations and deficits in reversal learning [48]. Of relevance, a decrease in the number of PV^+^ cells has been reported in many genetic ASD mouse models in various brain regions including mPFC, SSC and striatum; (see Table S1 in [48]). Also in the few human studies, a decrease in PV^+^ cells and/or *PVALB* mRNA was reported [46, 86]. Thus, the networks containing Pvalb neurons were hypothesized to be strongly implicated in ASD [45].

While an involvement of Pvalb neurons in ASD is rather undisputed, it remains unclear whether the observed reduction of PV^+^ cells in a particular brain region is the result of I) a truly decreased number of Pvalb neurons resulting from the many putative mechanisms including an immature or perturbed developmental state (e.g., layer- and/or region-inappropriate localization of Pvalb neurons, increased susceptibility, premature cell death, etc.) or II) alternatively from the down-regulation of PV protein levels or the failure to express adequate levels of the protein. To address this question, one needs to identify Pvalb cells by another means; one of the most common marker is the particular extracellular matrix surrounding Pvalb cells that can be visualized by VVA staining. Of note, the appearance of VVA staining is developmentally and layer-dependent regulated as shown in the mouse visual cortex (V1) [87] and the barrel cortex [88]. Moreover, PNNs are regulated by activity and are decreased under certain pathological conditions such as oxidative stress [89] or Alzheimer’s disease [90]. Thus, in the first step we ascertained by stereology [60] that the number of VVA^+^ cells was unchanged in mice with reduced (PV+/-) or absent (PV-/-) PV expression in the mPFC, SSC and striatum. In agreement with previous results obtained in the cortex and hippocampus of adult PV-/- mice [55, 57], there was no indication of a cell decrease/loss of Pvalb neurons. Moreover, in a mouse line expressing EGFP in Pvalb neurons, the number of EGFP^+^ cells was found to be the same as for PV^+^ and VVA^+^ cells in PV+/+ mice indicative of the identification of the essentially same Pvalb cell population, where either morphological or functional abnormalities have been reported in ASD [91–93]. Analyses of double-labeled (VVA^+^ and PV^+^) cells using either the total of PV^+^ or VVA^+^ cells for normalization revealed approximately 70–80 % of co-stained cells within the mPFC, SSC and striatum of WT mice. Similar numbers were reported in the mouse cortical V1 region, where 82 % of all VVA^+^ cells were found to also express PV [87]. While the percentage of PV^+^ cells enwrapped by PNN (VVA^+^) was not different between sections from WT and PV+/- mice, as the result of decreased PV protein expression levels in PV+/- mice, both the total number of PV^+^ cells, as well as the percentage of VVA^+^ cells expressing PV was significantly decreased in all regions analyzed in our study.

Thus, we wondered, whether the previously reported decrease in PV^+^ cells in other canonical ASD mouse models might not be -globally or in part- the result of PV down-regulation. We therefore assessed PV protein expression and VVA as a marker for Pvalb cells in the *Shank1* and *Shank3* mouse models. The selection among the many ASD mouse models available was based on the fact that more than 900 patients with genetic alterations in *SHANK* genes were identified, with the *SHANK* gene family being the primary gene family implicated in ASD [9]. *Shank1* and *Shank3* mouse models were selected to cover both extremes of the spectrum, namely *SHANK1* mutations found in individuals with ASD and normal intelligence and *SHANK3* mutations associated with severe mental retardation [9]. The selection was further facilitated by a recent comprehensive neuro-morphological MRI study on 26 ASD mouse models [75]. Similar to the most consistent finding in humans [94, 95], an increase in the frontal and parieto-temporal lobes and decreased volume of the cerebellar cortex were observed in one of the three subgroups (“group 1”) identified among the 26 investigated models [75]. A prominent example of the “group 1” ASD mouse models are *Shank3* null mutant mice. Importantly, however, also the morphological changes in PV-/- mice follow the same pattern, as we recently demonstrated: increased neocortical volume and a decreased size of the cerebellum [48]. A further link exists between Shank protein expression and Pvalb neurons [76, 96]. In *Shank1*-/- hippocampal PV^+^ FSI the absence of Shank1 functionally leads to a decrease in excitatory synaptic inputs and inhibitory synaptic outputs to pyramidal neurons and furthermore to molecular changes including the down-regulation of the postsynaptic proteins GKAP, PSD-95 and gephyrin [96]. These alterations affect the E/I balance in CA1 pyramidal neurons. Whether a similar situation prevails in *Shank1*-/- cortical PV^+^ FSI is currently unknown. In *Shank3* knockout mice, a reduction in PV^+^ puncta staining (intensity and puncta numbers) around pyramidal cells in the insular cortex of *Shank3B*-/- mice was associated with weakened GABAergic circuit function and impaired postnatal pruning [76]. Moreover, the decrease in PV-ir puncta intensity in *Shank3B*-/- hinted towards a PV expression-related phenomenon, although the question of PV expression levels was not directly addressed in this study.

For the *Shank* models our interest was focused on regions with high expression of either protein, i.e., SSC for *Shank1*-/- and striatum for *Shank3B*-/-. In both ASD models, the VVA^+^ cell number was unchanged and the number of PV^+^ cells was decreased, also evidenced by the decreased percentage of PV^+^ cells among all VVA^+^ cells. A decrease in PV protein levels and *Pvalb* mRNA levels to approximately 50 % of WT in both *Shank* mutants are in full support of a down-regulation of PV. In addition, the demonstration that PV levels are decreased may indicate a shift in the E/I balance towards an increased inhibition, taking into account the proven role of PV in synaptic transmission [52, 53, 55, 97, 98]. In striatal PV^+^ FSI the increased facilitation (increased inhibition) between FSI and medium spiny neurons (MSN) caused by the absence of PV in PV-/-mice [53] is partly compensated by a decrease in the excitatory synaptic input from cortical pyramidal cells, a mechanism hypothesized to compensate for the increased output of PV-/- neurons [48]. The modification of the E/I balance within the PV-circuitry in PV-/- mice is reminiscent of the situation in schizophrenia, where NMDA receptor hypofunction leads to the reduction of glutamic acid decarboxylase 67 (GAD67) levels and thus GABA synthesis. The concomitant decrease in PV expression levels might be viewed as an adaptive/compensatory response in order to enhance facilitation (inhibition) and to compensate –at least partially– for the decrease in GAD67 [99–101].

How might the absence of either Shank proteins lead to PV down-regulation? The similar magnitude in down-regulation of PV and *Pvalb* mRNA in both *Shank* mutants is indicative of a regulation at the transcriptional level. Currently little is known on the physiological regulation of PV expression in the brain. The enfeebled GABAergic circuit function reported in *Shank3B*-/- [76] and *Shank1*-/- mice [96] is likely to decrease somatic Ca^2+^ signals and subsequently modify the Ca^2+^ signaling components of Pvalb neurons; a neuronal activity-related Ca^2+^-dependent transcriptional regulation of PV expression was proposed before [100]. Thus, the impairment in GABAergic function reported in both *Shank* models is likely to impact on the Ca^2+^-dependent excitability-transcription (E/T) coupling including transcription of the *Pvalb* gene. Alterations in other genes implicated in E/T coupling have been observed before in ASD individuals and include Ca^2+^ signaling components such as the voltage-dependent channels Ca_v_1.2 (*CACNA1C*), Ca_v_1.3 (*CACNA1D*) and the α-δ auxiliary subunit of L-type voltage-gated Ca^2+^ channels (*CACNA2D3*) [102, 103]. Evidence has accumulated that mutated ASD risk genes are critical components of activity-regulated signaling networks often controlling synapse development and morphology, as well as structural and functional plasticity [8]. In summary, the observed decrease in PV expression in *Shank* mutant mice might be viewed as an adaptive or compensatory mechanism to possibly restore (increase) synaptic output according to the concept of the Ca^2+^ homeostasome [104].

Our findings might have important implications for novel treatment strategies for ASD, particularly as most current strategies aim to enhance inhibition in order to compensate for a presumed increase in excitatory neurotransmission, e.g., by the GABA_B_ receptor agonist barbaclofen [80, 105]. Our findings indicate the exact opposite, namely that an enhancement of excitatory neurotransmission onto PV^+^ neurons possibly restoring PV levels and thus PV-modulated functions (e.g., short-term plasticity, synchronization, precision of spike timing) may ameliorate ASD symptoms. Alternatively, one might envisage that direct up-regulation of PV might be a means to ameliorate PV-circuitry function resulting in the attenuation -or in the best case abolition- of the ASD phenotype.

## Conclusions

Stereological analysis of Pvalb neurons in mice heterozygous and homozygous for a deletion of the functional *Pvalb* gene (PV+/- and PV-/- mice, respectively, both showing an ASD-like phenotype) revealed their numbers to be unaltered in comparison to WT mice. A similar situation as in PV+/- mice prevailed in the two ASD mouse models *Shank1*-/- and *Shank3B*-/-: the number of Pvalb neurons was unchanged in brain regions with high expression of either protein, while PV protein and *Pvalb* mRNA levels were decreased by approximately 50 %. Based on the similar magnitude in PV down-regulation and impairment in the E/I balance reported before in all three models we hypothesize that the PV system, in particular reduced PV expression levels, might represent a convergent downstream endpoint for some forms of ASD.

### Ethics approval and consent to participate

All experiments were performed with permission of the local animal care committees (Canton of Fribourg, Switzerland; Regierungspräsidium Gießen, Germany) and according to the present Swiss/Germany law and the European Communities Council Directive of 24 November 1986 (86/609/EEC).

## Abbreviations

ASD: autism spectrum disorders
CB: calbindin D-28k
CE: coefficient of error
EGFP: enhanced green fluorescent protein
FSI: fast-spiking interneurons
GAD67: glutamic acid decarboxylase 67
ISH: in situ hybridization
mPFC: medial prefrontal cortex
PND: postnatal day
PV: parvalbumin
SSC: somatosensory cortex
VVA: vicia villosa agglutinin
